# Stimulation of Mononuclear Cells Through Toll-Like Receptor 9 Induces Release of Microvesicles Expressing Double-Stranded DNA and Galectin 3-Binding Protein in an Interferon-α-Dependent Manner

**DOI:** 10.3389/fimmu.2019.02391

**Published:** 2019-10-11

**Authors:** Niclas Stefan Rasmussen, Christoffer Tandrup Nielsen, Søren Jacobsen, Claus Henrik Nielsen

**Affiliations:** ^1^Center for Rheumatology and Spine Diseases, Institute for Inflammation Research, Copenhagen University Hospital, Rigshospitalet, Copenhagen, Denmark; ^2^Copenhagen Lupus and Vasculitis Clinic, Center for Rheumatology and Spine Diseases, Copenhagen University Hospital, Rigshospitalet, Copenhagen, Denmark

**Keywords:** peripheral blood mononuclear cells, T cells, microvesicles, Toll-like receptor 9 agonist, type 1 interferon, galectin-3 binding protein, dsDNA, systemic lupus erythematosus

## Abstract

**Background:** Microvesicles (MVs) expressing the type 1 interferon (IFN)-inducible protein galectin-3 binding protein (G3BP) may play a pathogenic role in systemic lupus erythematosus (SLE). Co-expression of double-stranded DNA (dsDNA) on such MVs may render them immunogenic and targets for anti-dsDNA antibodies. Little is known about the mechanisms underlying generation of this MV population. In this study, we investigated how Toll-like receptors (TLRs), IFN-α, and T cells are involved in this process in healthy subjects.

**Methods:** Peripheral blood mononuclear cells (PBMCs) isolated from 12 healthy donors were stimulated *in-vitro* for 24 h with a series of TLR-agonists or the T cell activating antibody OKT3 or were subjected to apoptosis by incubation with staurosporine. MVs in the supernatants were subsequently isolated by differential centrifugation and were quantified and characterized with respect to expression of G3BP and dsDNA by flow cytometry.

**Results:** Stimulation of PBMCs with the TLR9-agonist and strong IFN-α inducer ODN2395 significantly increased the release of MVs expressing G3BP. The production of MVs with this phenotype was markedly enhanced by co-stimulation of T cells. Furthermore, dependency on IFN-α in the generation of G3BP-expressing MVs was indicated by a marked reduction following addition of the IFN-α inhibitor IFN alpha-IFNAR-IN-1 hydrochloride.

**Conclusion:** Release of G3BP-expressing MVs from healthy donor PBMCs is induced by stimulation of TLR9 in an IFN-α-dependent manner and is enhanced by co-stimulation of T cells.

## Background

Extracellular vesicles (EVs) are double-layered membrane vesicles that may be released by cells in response to activation or during apoptosis (1, 2). Accumulating evidence shows that EVs are not inert, but usually carry an orchestrated cargo of proteins and nucleic acids with diverse physiological roles in health and disease (3, 4). These include diverse paracrine functions, with extracellular RNAs playing a central role (5), but it is recognized that also other mechanisms should be explored (6). There is a growing interest and understanding of the role of EVs in the context of systemic autoimmune disease, in particular systemic lupus erythematosus (SLE) (7).

EV studies in SLE have mainly focused on so-called microparticles that comprise 0.1–1 μm microvesicles (MVs) and the somewhat larger apoptotic bodies (8). SLE MVs carry double-stranded DNA (dsDNA), whereby they may become targets for binding of anti-dsDNA antibodies (9). Proteomics show that circulating MVs from SLE patients hold a characteristic signature of increased expression of galectin-3 binding protein (G3BP), immunoglobulin G (IgG), and several other proteins (10, 11). Complementary to these findings are flow cytometric analyses showing elevated blood levels of MVs with surface-bound IgG (12) or G3BP (13) in SLE patients. The origin and pathogenic relevance of this subset of MVs remain obscure. However, defective removal of cellular remnants and immune complexes (ICs) are well-established elements of SLE pathogenesis (14), and specific roles of dsDNA-loaded MVs in this context have been suggested (15, 16). In SLE patients with nephritis, deposits of IgG in the glomerular basement membrane (GBM) colocalize with chromatin forming electron dense structures (EDS) (17) that also contain G3BP (13).

G3BP is a type 1 interferon (IFN)-inducible protein that belongs to the scavenger receptor cysteine-rich (SRCR) superfamily (18) and binds to several components of the GBM, including nidogen, collagen IV, and fibronectin (19)–a property which, in theory, renders G3BP-expressing MVs glomerulophilic.

The increased production of IFN-α frequently found in SLE patients with active disease is thought to prime the immune system toward breach of self-tolerance and persistent autoimmune reactions and appears to be linked to Toll-like receptor (TLR)7 and TLR9 ligation by nucleic acid-containing ICs (20). The notion has been carried forward that ICs are presented to the immune system in the context of chromatin-loaded vesicles formed during apoptosis (16), but the role of smaller MVs formed during cellular activation has not been investigated.

Given that G3BP is type 1 IFN-inducible (21), we speculated that MVs expressing dsDNA and G3BP may be released from mononuclear cells as a result of TLR- and type 1 IFN-mediated activation. Further, since activated T cells may enhance TLR-mediated IFN-α production (22), T cells may play a particular role in this type of MV generation.

In this study, we tap into this hypothesis by stimulating peripheral blood mononuclear cells (PBMCs) from healthy subjects with different TLR ligands, a T cell stimulator and a type-1 IFN inhibitor to quantify the generation of dsDNA- and G3BP-expressing MVs.

## Materials and Methods

### Blood Donors

Blood from anonymous healthy donors was obtained from the Blood Bank at Copenhagen University Hospital, Rigshospitalet. The study was approved by the Scientific-Ethical Committee of the Capital Region of Denmark (protocol no. H-15004075).

### Isolation and Staining of Platelets

Three milliliter of blood was collected in Multiplate® Hirudin blood tubes (Roche Diagnostics GmbH, Mannheim, Germany) by venous puncture. The blood was centrifuged at 1,800 × g for 10 min at 21°C, platelet-rich plasma was carefully aspirated, and 1 mL was transferred to Eppendorf tubes (Corning, New York City, USA) and centrifuged at 3,000 × g for 10 min at 21°C to pellet platelets. The platelet-poor plasma was carefully aspirated and discarded, and platelets were gently resuspended in 300 μL phosphate-buffered saline (PBS) (Thermo Fisher Scientific, Waltham, USA) filtered through a 0.2 μm filter (Sartorius, Göttingen, Germany). Five microliter of the platelet-isolate was pipetted into 37.5 μL filtered PBS in FACS tubes (Corning) followed by addition of 5 μL APC-conjugated anti-CD61 antibody (BD, Franklin Lakes, USA) and 2.5 μL calcein-acetoxymethyl ester (calcein-AM) (Sigma-Aldrich, St. Louis, USA) (2.5 μg/ml in filtered PBS) to a final volume of 50 μL. The tubes were incubated for 1 h at room temperature (RT) in the dark. After incubation, 100 μL TruCount beads (BD) and 125 μL Megamix-Plus side-scatter (SSC) beads (Biocytex, Marseille, France) were added to the tubes. The volume was adjusted to 300 μL with filtered PBS before analysis. The TruCount bead solution was prepared by dissolving the lyophilized beads in 500 μL filtered PBS. Samples were analyzed on a FACSCanto II flow cytometer (BD) at low flow-rate and with lowest SSC threshold (=200).

### Isolation of PBMCs

Blood was collected in Vacutainer® EDTA tubes (Greiner Bio-one GmbH, Kremsmünster, Austria) by venous puncture and centrifuged at 1,800 × g for 10 min at 21°C. Platelet-rich plasma was aspirated, and the PBMCs were poured onto a density gradient medium (Lymphoprep™; Alere Technologies, Oslo, Norway), centrifuged at 1,172 × g for 30 min at 24°C, washed twice in sterile PBS, and finally resuspended in sterile medium consisting of RPMI-1640 GlutaMAX medium (Lonza, Basel, Schweiz) supplemented with 20% heat-inactivated fetal calf serum (hFCS) (Sigma-Aldrich) and 0.1% gentamicin (BI, Kibbutz Beit Haemek, Israel). PBMCs were subsequently counted using the NucleoCounter® NC-100™ system (ChemoMetec, Allerød, Denmark) according to the manufacturer's instructions. The PBMC-isolate was divided into 500 μL aliquots in cryotubes (DACOS, Esbjerg, Denmark), followed by addition of 500 μL sterile medium supplemented with 30% hFCS and 20% dimethylsulfoxid (DMSO) (Merck kGaA, Darmstadt, Germany), yielding a final concentration of 25% hFCS and 10% DMSO. The cryotubes were inverted, placed in CoolCell® freezing containers (BioCision, San Rafael, USA), and stored at −80°C for at least 24 h, before they were cryopreserved.

### Stimulation of PBMCs

The cryopreserved PBMCs were thawed at RT, washed and resuspended in sterile medium supplemented with 20% hFCS (Sigma-Aldrich) and 0.1% gentamicin (BI). Their viability was confirmed using the NucleoCounter® NC-100™ system according to the manufacturer's instructions. The cells were plated into 48-well plates with UpCell™ surface (Nunc, Roskilde, Denmark) at ~600,000 PBMCs per well and were rested for 30 min at 37°C and 5% CO_2_ before incubation for 24 h at 37°C and 5% CO_2_ with the following components or combinations hereof: staurosporine for induction of apoptosis (Abcam, Cambridge, UK) (2.5 μM); the TLR3-agonist poly(A:U) (Invivogen, San Diego, USA) (20 μg/mL); the TLR4-agonist lipopolysaccharide (LPS) (Invivogen) (1.25 μg/mL); the TLR7-agonist gardiquimod (Invivogen) (1.5 μg/mL); the TLR9-agonists ODN2006 (Invivogen) (12 μg/mL) or ODN2395 (Invivogen) (12 μg/mL) (23); the inhibitor of the interaction between IFN-α and the IFN-α receptor (IFNAR) IFN alpha-IFNAR-IN-1 hydrochloride (IN-1) (MedchemExpress, Sollentuna, Sweden) (32 μM) (24); the T cell stimulating anti-CD3 antibody OKT3 (Invitrogen, Carlsbad, USA) (1 μg/mL) (25).

### Preparation of Culture Supernatants

After incubation with stimuli the plates were left at RT for 15 min. Adhered cells were gently loosened and transferred to FACS tubes. The cell suspensions were centrifuged at 458 × g for 10 min at 24°C to pellet PBMCs. The cell-free supernatants were then harvested, aliquoted into cryotubes, and snap-frozen in liquid nitrogen. Samples were stored at −80°C until analysis.

### Isolation of MVs From Culture Supernatants

The frozen cell-free supernatants were thawed at RT, transferred to Eppendorf tubes, and centrifuged at 3,000 × g for 10 min at 21°C to pellet larger particles and potential cell residues. The supernatants were aspirated down to 50 μL and transferred to new tubes (200 μL in each). For some experiments, DNase solution (Stemcell Technologies, Vancouver, Canada) was added to the tubes to a final concentration of 0.1 mg/ml (~200 U/mL) and the tubes were then incubated for 1 h at 37°C and 5% CO_2_. The samples were ultracentrifuged at 20,000 × g for 30 min at 21°C to pellet MVs. Subsequently, 175 μL supernatant was aspirated and discarded, and MVs were then resuspended in 175 μL PBS filtered through 0.2 μm pores, followed by another ultracentrifugation step. The supernatant was aspirated as before and discarded, and MVs were resuspended in 70 μL filtered PBS to a final volume of 95 μL (MV-isolate).

### Detection and Staining of MVs

We used calcein as a general marker of MVs (26). Any Fcγ-receptors on MVs were blocked by adding 5 μL commercial Fc blocker (BD) to MV-isolates for 15 min at RT. After this incubation, 5 μL MV-isolate was pipetted into 22.5 μL filtered PBS in FACS tubes followed by addition of 5 μL mouse anti-human G3BP antibody of IgG2b isotype (clone: 2D8E11) (Proteintech, Manchester, UK) (1 μg/mL in filtered PBS) or isotype control (clone: MG2b-57) (Biolegend, San Diego, USA), 5 μL mouse anti-dsDNA antibody of IgG2a isotype (clone: HYB 331-01) (SSI, Copenhagen, Denmark) (0.5 μg/mL in filtered PBS), or isotype control (clone: MG2a-53) (Biolegend). Next, 5 μL APC-conjugated goat anti-mouse IgG2b antibody (Southern Biotech, Birmingham, USA) (0.5 μg/mL in filtered PBS), 5 μL BV510-conjugated rat anti-mouse IgG2a antibody (BD) (0.5 μg/mL in filtered PBS), and 2.5 μL calcein-AM (2.5 μg/mL in filtered PBS) were added, yielding a final volume of 50 μL. Unstained and single-stained controls were included. The tubes were incubated for 1 h at RT in the dark. After incubation, 100 μL TruCount beads (BD) were added to the tubes and the volume was adjusted to 300 μL with filtered PBS before acquisition on a FACSCanto II flow cytometer (BD) at low flow-rate and with lowest SSC threshold (=200). The TruCount bead solution was prepared by dissolving the lyophilized beads in 500 μL filtered PBS. Each sample was run for 4 min or until a minimum of 1,000 TruCount bead events were recorded.

### Quantification and Size Determination of MVs

The absolute count of MVs (MVs/μL) was calculated with TruCount beads as reference, using the formula:

[[(no. of MV events within gates of interest)/(no. of collected bead events)] × [(total no. of beads)/(test volume)]] × (dilution factor).

Megamix-Plus SSC beads–a SSC optimized mixture of polystyrene beads with size references of 0.16, 0.2, 0.24, and 0.5 μm–were utilized to define a SSC specified MV gate. Due to the higher refractive index (RI) of polystyrene relative to that of MVs (lipid vesicles), these size references are not directly translatable, as described by van der Pol et al. and others (27–30). To allow for such discrepancy, we estimated lipid vesicle equivalents based on Mie theory (31–33) by taking the RI of the particles and surrounding medium, the collection angle of the scattered light, and the illumination wavelength and intensity into account. All estimations were made with the free software Mieplot (www.philiplaven.com/mieplot.htm). The resulting plots (Supplementary Figure 1) depict that the SSC light of 0.2 and 0.24 μm polystyrene beads corresponds to that of 0.5 and 1.0 μm lipid vesicles, respectively, and the SSC light of 0.5 μm polystyrene beads corresponds to that of 2.7 μm lipid vesicles.

### Transmission Electron Microscopy

MV-isolates were adsorbed onto carbon-coated grids for 1 min. The excess liquid was removed with filter paper, and the grids were then washed in double-distilled water prior to staining with 3% uranyl acetate solution for 1 min. Using the principle of negative staining, the samples were analyzed on a CM100 transmission electron microscope (Philips, Eindhoven, Netherlands).

### Statistical Analysis

Wilcoxon signed-rank test was used for comparisons of concentrations and ratios between paired samples. In cases where the background (non-stimulated controls) was subtracted, the test was used to assess if the net values differed from zero. The statistical analysis was performed in GraphPad Prism software 8 (GraphPad Software Inc., San Diego, USA). *P* < 0.05 were considered statistically significant.

## Results

### Flow Cytometric Measurement of MVs

**Figure 1:**
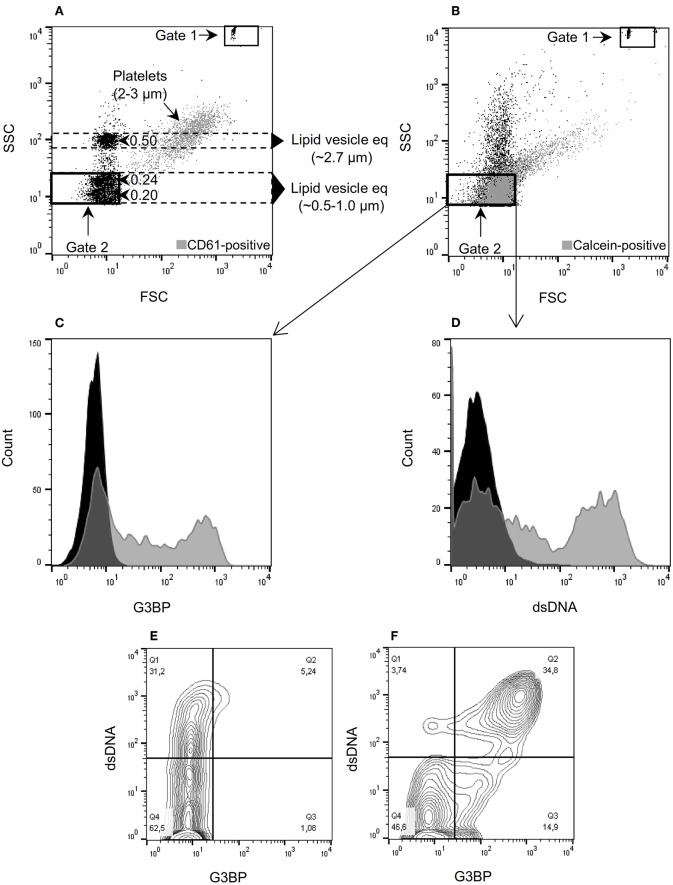
Microvesicle staining, gating, and quantification. **(A)** Megamix-Plus side-scatter (SSC) beads (polystyrene) with size references of 0.16, 0.2, 0.24, and 0.5 μm were applied to define a flow cytometric SSC-based gate for microvesicles (MVs) (defined as lipid vesicles). Lipid vesicle equivalents (eq) of the indicated sizes were estimated, taking the different refractive indices of polystyrene and lipid vesicles into account. Gate 1 contains TruCount beads used for quantification of MVs. Gate 2 corresponds to the MV gate used throughout the study. For comparison, normal platelets stained with anti-CD61 antibody were added to the sample (gray events). **(B)** Forward-scatter (FSC)/SSC characteristics of MVs isolated from culture supernatants and stained with calcein (gray events). **(C)** MVs contained in culture supernatants from peripheral blood mononuclear cells (PBMCs) incubated with the TLR9-agonist ODN2395. The MVs were incubated with calcein and anti-G3BP antibody (gray) or isotype control (black). **(D)** Corresponding histogram after staining with anti-dsDNA antibody (gray) or isotype control (black). **(E)** Contour plot of MVs released from non-stimulated PBMCs and stained for G3BP (x-axis) and dsDNA (y-axis). **(F)** Corresponding contour plot of MVs released from PBMCs stimulated with ODN2395. Events within gate 2 are shown in **(C)** through **(F)**.

MVs isolated from a total of 12 healthy donors [10 women and 2 men, median age 26 years (range 22–63)] were analyzed using the flow cytometry gating shown in Figure 1. TruCount beads (Figures 1A,B, gate 1) were used for quantification and Megamix-Plus SSC beads (0.16, 0.2, 0.24, and 0.5 μm) were used as size reference to define the MV gate (Figures 1A,B, gate 2). Normal platelets (Figure 1A, gray events) of ~2–3 μm in diameter (34) served to validate the estimated lipid vesicle equivalents. In agreement with the predictions, the SSC light of platelets showed considerable overlap with the 0.5 μm bead population (Figure 1A, y-axis). The majority of detectable calcein-positive events in the differentially centrifuged culture supernatants (Figure 1B, gate 2, gray events) localize within the predicted MV gate, supporting that most of these events are in the size range of MVs. Treatment of culture supernatants with detergent (1% Triton X-100) prior to isolation of MVs abolished the signal from calcein, confirming the lipid nature of calcein-positive events (Supplementary Figure 2A). Moreover, MVs were only detectable in supernatants from setups containing added PBMCs, confirming that the signal from calcein within the MV gate is derived completely from the experimental cells and not from artifacts or residual MVs potentially present in the hFCS (Supplementary Figure 2B). We also evaluated the MV assay for coincident events which might cause false colocalization signals, but such phenomenon was not observed (Supplementary Figure 2C).

### Identification of MVs With Transmission Electron Microscopy

**Figure 2:**
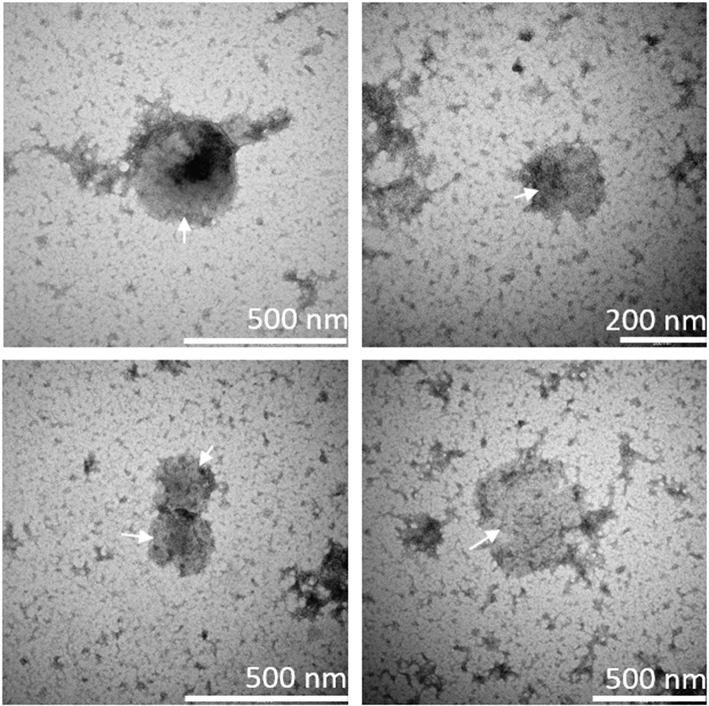
Presence of microvesicles in culture supernatant. Microvesicles (MVs) in culture supernatant from ODN2395-stimulated peripheral blood mononuclear cells were visualized by means of transmission electron microscopy using the negative stain principle. Arrows indicate round-shaped particles within the size range of MVs as indicated by the scale bars in the lower right corners.

The presence of MVs in culture supernatants was confirmed by use of transmission electron microscopy. Specifically, culture supernatants from ODN2395-stimulated PBMCs were investigated; round-shaped particles within the MV-size range were identified, showing the presence of MVs in these supernatants (Figure 2).

### TLR-Mediated Release of dsDNA- and G3BP-Expressing MVs From Mononuclear Cells

Stimulation of PBMCs with the TLR9-agonist ODN2395 lead to release of MVs with distinct expression of G3BP (Figure 1C) and surface-bound dsDNA (Figure 1D) into the culture supernatants. Notably, about 1/3 of MVs present in supernatants from unstimulated PBMCs bore dsDNA but not G3BP (Figure 1E). Stimulation with ODN2395 induced co-expression of G3BP (Figure 1F).

**Figure 3:**
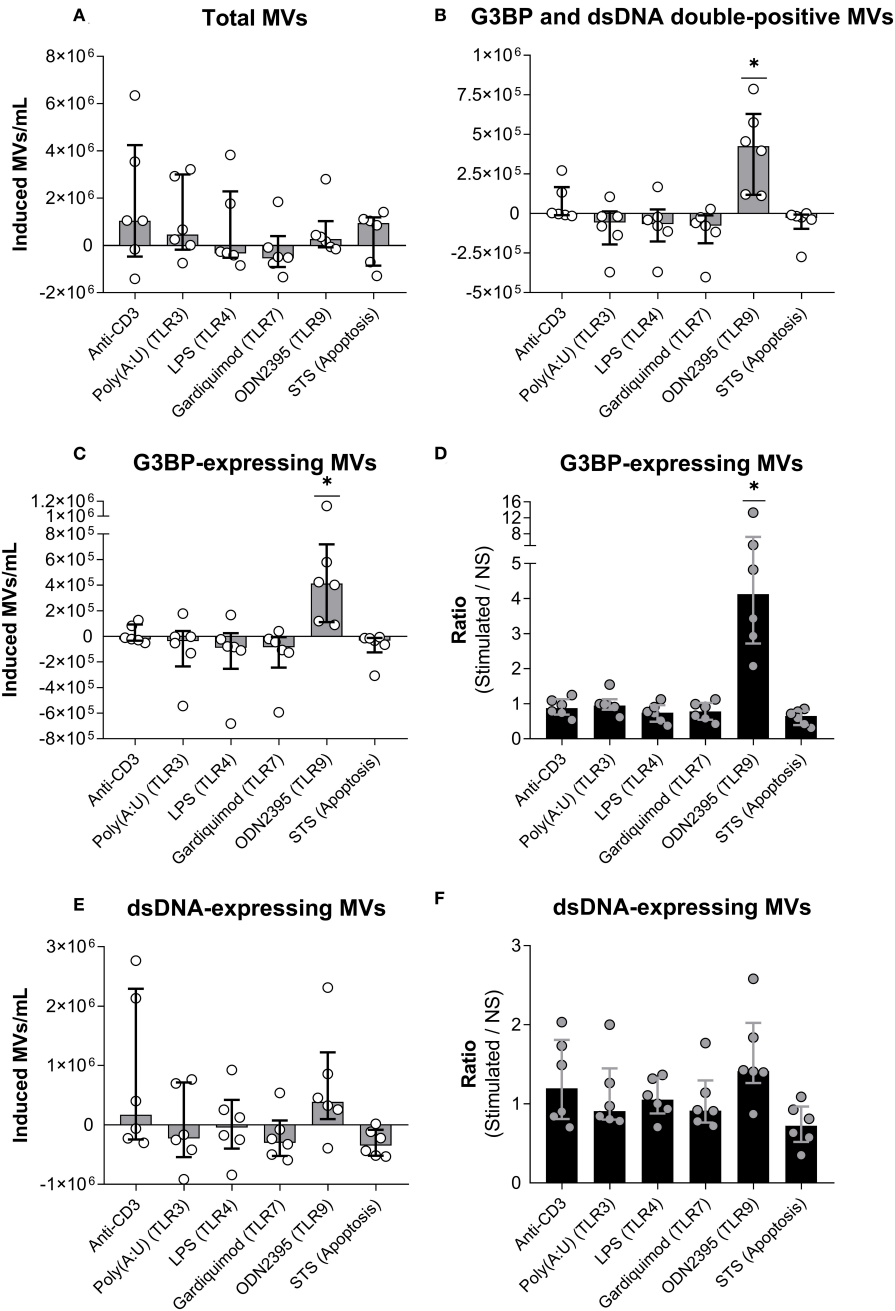
Release of G3BP- and/or dsDNA-expressing microvesicles from peripheral blood mononuclear cells. **(A)** Peripheral blood mononuclear cells from healthy donors were incubated for 24 h with either anti-CD3 antibody (OKT3) (*n* = 6), the TLR3-agonist poly(A:U) (*n* = 6), the TLR4-agonist LPS (*n* = 6), the TLR7-agonist gardiquimod (*n* = 6), or the TLR9-agonist ODN2395 (*n* = 6), or were treated with staurosporine (STS) to induce apoptosis (*n* = 6). Microvesicles (MVs) released into the culture supernatants were subsequently isolated by differential centrifugation. Isolated MVs were quantified and characterized with respect to expression of G3BP and dsDNA by flow cytometry. The change in the concentration of MVs in culture supernatants, induced by the various stimuli, is shown. Columns and error bars represent median values and interquartile range after subtraction of background (non-stimulated controls). **(B)** Corresponding quantifications of G3BP and dsDNA double-positive MVs. **(C)** Changes in the concentration of the G3BP-expressing MV population *in toto*. **(D)** The fold change in the concentration of G3BP-expressing MVs, expressed as the ratio of MV count in stimulated samples over that of non-stimulated samples (NS). **(E)** Changes in the concentrations of the dsDNA-expressing MV population *in toto*. **(F)** The fold change in the concentration of dsDNA-expressing MVs. *P < 0.05. NS, non-stimulated.

We stimulated PBMCs with a series of TLR-agonists or the T cell activating antibody OKT3, or induced apoptosis by incubation with staurosporine (Figure 3). None of the stimuli significantly affected the total number of MVs released, as shown in Figure 3A, where the number of MVs in non-stimulated cultures have been subtracted (allowing occurrence of negative values). However, as the only stimulus, ODN2395 induced a significant increase in the number of G3BP and dsDNA double-positive MVs (Figure 3B) and, accordingly, in the total number of G3BP-expressing MVs (Figure 3C). The content of G3BP-expressing MVs was thus a median of four times higher in OD2395-stimulated cultures than in non-stimulated cultures (Figure 3D). None of the other stimuli examined affected the release of G3BP-expressing MVs.

Stimulation with ODN2395 induced a non-significant increase in the number of dsDNA-expressing vesicles released into the supernatant (Figures 3E,F). Specificity of the staining for dsDNA was confirmed by the observation that preincubation of culture supernatants from ODN2395-treated PBMCs with DNase markedly reduced the binding of the detecting anti-dsDNA antibody to MVs (data not shown).

### Influence of T Cells on TLR9-Mediated Release of MVs

**Figure 4:**
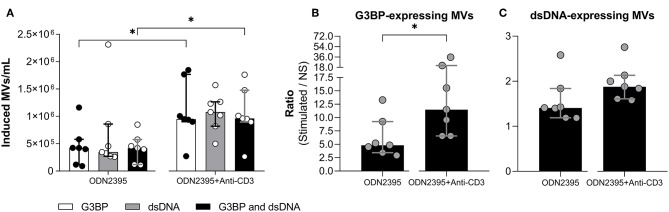
Effect of T cell stimulation on release of microvesicles from peripheral blood mononuclear cells. Peripheral blood mononuclear cells from healthy donors (*n* = 7) were incubated for 24 h with the TLR9-agonist ODN2395 in presence or absence of anti-CD3 antibody (OKT3). Microvesicles (MVs) released into the culture supernatants were subsequently isolated by differential centrifugation. Isolated MVs were quantified and characterized with respect to expression of G3BP and dsDNA by flow cytometry. **(A)** Concentration of MVs expressing G3BP (white columns), dsDNA (gray columns), or both (black columns) after subtraction of concentrations observed in non-stimulated cultures. **(B)** The corresponding fold change, expressed as the ratio between G3BP-expressing MVs in supernatants from stimulated and non-stimulated cultures (NS). **(C)** The corresponding fold change in the dsDNA-expressing MV population. Columns and error bars represent median values and interquartile range. *P < 0.05. NS, non-stimulated.

As indicated above, cross-binding of CD3 on T cells *per se* did not influence MV release from mononuclear cells. To investigate whether cross-binding of CD3 had any influence on the TLR9-mediated MV release, we stimulated PBMCs with ODN2395 alone or in combination with anti-CD3 antibody (Figure 4). The co-stimulation markedly enhanced the ODN2395-induced release of G3BP-expressing MVs *in toto* and of G3BP and dsDNA double-positive MVs (Figures 4A,B), whereas the release of dsDNA-expressing MVs *in toto* was not affected significantly (Figures 4A,C).

### Effect of Different TLR9-Agonists on MV Release and Phenotype

**Figure 5:**
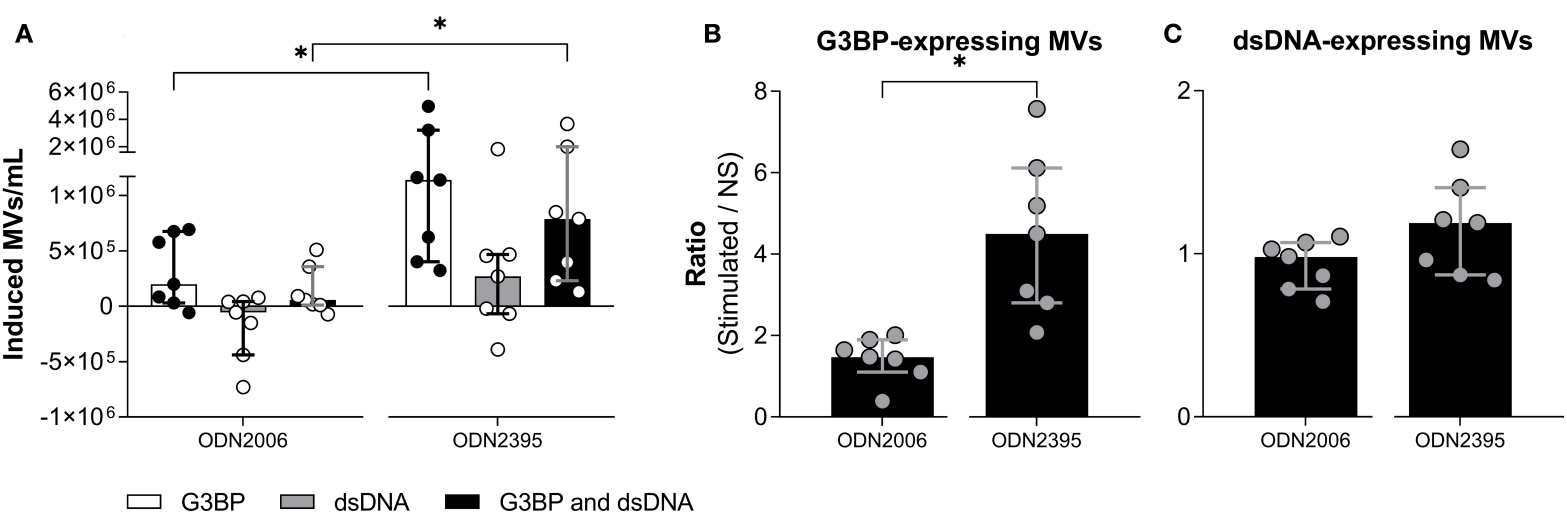
Effect of different TLR9-agonists on release of microvesicles from peripheral blood mononuclear cells. Peripheral blood mononuclear cells from healthy donors (*n* = 7) were incubated for 24 h with ODN2006, a weak inducer of IFN-α, or ODN2395, a strong inducer of IFN-α. Microvesicles (MVs) released into the culture supernatants were subsequently isolated by differential centrifugation. Isolated MVs were quantified and characterized with respect to expression of G3BP and dsDNA by flow cytometry. **(A)** Shown is the consequent increase in the concentration of MVs expressing G3BP (white columns), dsDNA (gray columns), or both (black columns). **(B)** The concentration of G3BP-expressing MVs in cultures of stimulated cells relative to that in non-stimulated cultures (NS). **(C)** The corresponding relative concentration of dsDNA-expressing MVs. Columns and error bars represent median values and interquartile range. *P < 0.05. NS, non-stimulated.

Since ODN2395 is a potent inducer of IFN-α, we examined how the effect of another TLR9-agonist, ODN2006, which is known to be a weak IFN-α-inducer (23), affected MV release and phenotype studied. Notably, ODN2006 was a much weaker stimulus for release of G3BP-expressing MVs than ODN2395 (Figures 5A,B), suggesting that acquisition of this phenotype depended, at least in part, on secretion of IFN-α. The release of dsDNA-expressing MVs *in toto* did not differ significantly between the two stimuli (Figures 5A,C).

### Effect of IFN-α Inhibition on TLR9-Induced Release of MVs

**Figure 6:**
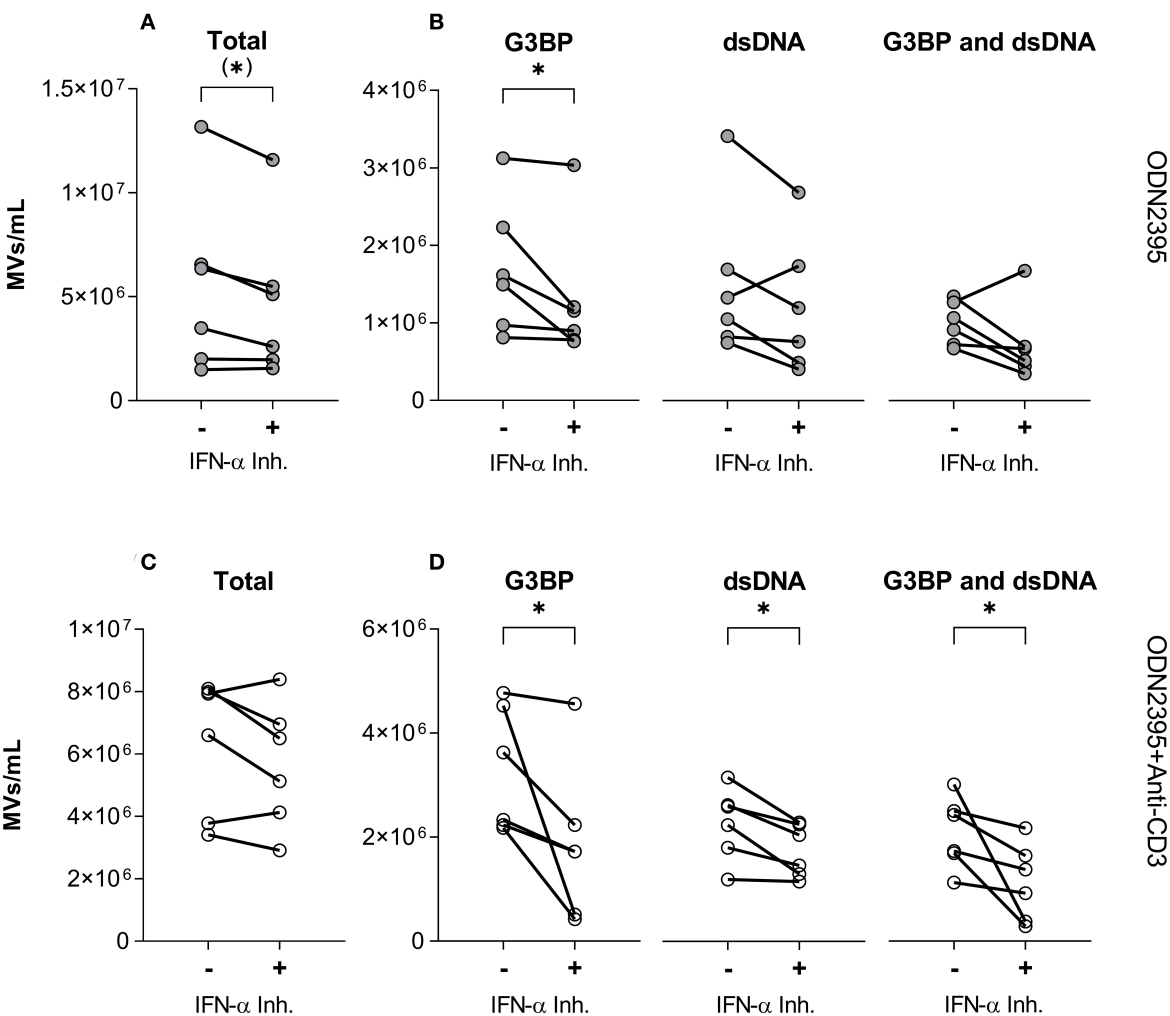
Effect of IFN-α inhibition on TLR9-induced release of microvesicles from peripheral blood mononuclear cells. Peripheral blood mononuclear cells from healthy donors (*n* = 6) were incubated for 24 h with the TLR9-agonist ODN2395, alone (–) or in combination with the IFN-α inhibitor IFN alpha-IFNAR-IN-1 hydrochloride (+). Released microvesicles (MVs) were quantified, and expression of G3BP and dsDNA by the MVs was analyzed by flow cytometry. **(A)** Concentration in the culture supernatant of the total MV population and **(B)** subpopulations of G3BP-expressing, dsDNA-expressing, and G3BP and dsDNA double-positive MVs. **(C)** Corresponding concentration of the total MV population and **(D)** subpopulations after co-stimulation of T cells with anti-CD3 antibody. *P < 0.05.

To test directly if IFN-α was involved in generation of G3BP- or dsDNA-expressing MVs, we employed the IFN-α inhibitor IN-1 (Figure 6). Despite having little effect on the overall release of MVs from PBMCs stimulated with ODN2395 (Figure 6A), this inhibitor significantly reduced the release of G3BP-expressing MVs (Figure 6B). A similar effect pattern was observed after co-stimulation of the PBMCs with anti-CD3 antibody (Figures 6C,D), but in this situation the release of dsDNA- and double-positive MVs was also reduced significantly by IN-1 (Figure 6D).

## Discussion

The purpose of this study was to investigate the mechanisms underlying release of MVs from normal mononuclear cells, and to characterize the released MVs with respect to expression of dsDNA and G3BP. Insight into these mechanisms may enhance our understanding of MV release, in general, and, since G3BP-expressing MVs may deposit on the GBM, it may also help to understand how nephritis develops in SLE (35).

Our main finding was that incubation of PBMCs with the TLR9-agonist ODN2395 caused qualitative changes in MVs released from cultivated PBMCs, while the total number of MVs released were largely unchanged. Thus, ODN2395 induced a substantially increased co-expression of G3BP and dsDNA on the MV surface. A surface-localized signal from both the former and latter is supported by the vesicles' ability to retain calcein, suggesting low vesicular permeability. By contrast, the TLR3-agonist poly(A:U), the TLR4-agonist LPS and the TLR7-agonist gardiquimod had no effect on the total MV release, nor on the MV phenotypes studied. TLR9 binds hypomethylated CpG-rich DNA (36), suggesting that ICs containing such DNA may be a physiological stimulus for release of G3BP-expressing MVs. However, the TLR9-agonist ODN2006 did not induce release of G3BP-expressing MVs. This discrepancy may be related to the ability of ODN2395 to induce production of IFN-α in contrast to ODN2006 (23). In accordance with this notion, we observed a marked reduction in the frequency of G3BP-expressing MVs in presence of the IFN-α inhibitor IN-1. The enhancement of MV expression of G3BP by IFN-α may be relevant to SLE pathogenesis, in light of the exaggerated production of IFN-α by plasmacytoid dendritic cells (pDCs) in this disease (20).

In contrast with the markedly increased G3BP-expression by MVs after stimulation with ODN2395, this TLR-agonist had little effect on the expression of dsDNA by the MVs; nor was this expression differentially regulated by the two TLR9-agonists used in this study.

To examine the effect of T cell stimulation on MV-production and phenotype, we included an anti-CD3 antibody as stimulus. Interestingly, this stimulus markedly enhanced the ODN2395-induced generation of G3BP-expressing MVs and of G3BP and dsDNA double-positive MVs. These effects of T cells may rely on cytokine production by the T cells, in keeping with previous findings that *in-vitro* activated T cells from healthy donors and SLE patients enhance the secretion of IFN-α from pDCs stimulated with the TLR9-agonist ODN2216 (22). However, T cell TLR9 and the T cell receptor (with CD3 as co-receptor) have previously been demonstrated to act in concert (37) and we cannot exclude that the increased number of G3BP-expressing MVs released following co-stimulation via CD3 originate from T cells *per se*. We have previously shown that a significant proportion of MVs isolated from the blood of healthy donors and SLE patients express CD3, indicating that they have been released by T cells (38).

G3BP has been shown to bind to several proteins, including collagen IV, nidogen, and fibronectin (19), all of which have been demonstrated in the GBM. It also binds to galectin-1 and galectin-3 with high affinity (39), and both these galectins are expressed by many immune cells, including T cells, B cells and macrophages (40, 41). Under physiological circumstances, expression of G3BP by MVs may therefore serve an immunoregulatory function. Moreover, G3BP has a scavenging function and may thus facilitate clearance of MVs (18). In SLE, however, G3BP-expressing MVs may deposit in kidney glomeruli, where overexpression of galectin-3 has been observed (42). The dsDNA co-expressed by the MVs is likely to become target for anti-dsDNA antibodies and complement activation may ensue. To this end, bound IgG (12) and complement fragments (38) have been demonstrated on circulating MVs from SLE patients. It has thus been speculated that G3BP and dsDNA double-positive MVs may deposit in the GBM and contribute to the kidney damage observed in SLE (8).

In conclusion, we show that stimulation through TLR9 induce G3BP-expression of MVs released from healthy donor PBMCs in an IFN-α-dependent manner, and that a substantial proportion of the MVs co-express dsDNA. The excessive production of IFN-α and anti-dsDNA antibodies in SLE and overexpression of galectin-3 in the patients' kidney glomeruli suggest that G3BP and dsDNA co-expressing MVs hold a strong pathogenic potential in this disease.

## Ethics Statement

The study was approved by the Scientific-Ethical Committee of the Capital Region of Denmark (protocol no. H-15004075).

## Author Contributions

SJ, CTN, and CHN created the research concept and supervised the research and the preparation of the manuscript. NR, CTN, SJ, and CHN designed the research. NR conducted the experiments, analyzed data, and wrote the manuscript.

### Conflict of Interest

The authors declare that the research was conducted in the absence of any commercial or financial relationships that could be construed as a potential conflict of interest.
