# Supplementary material for: Stimulation of Mononuclear Cells Through Toll-Like Receptor 9 Induces Release of Microvesicles Expressing Double-Stranded DNA and Galectin 3-Binding Protein in an Interferon-α-Dependent Manner
Source: Front Immunol. 2019 Oct 11;10:2391. doi: 10.3389/fimmu.2019.02391 (PMC6797593; doi:10.3389/fimmu.2019.02391)
Supplement: Supplementary file 1 [file Data_Sheet_1.pdf]

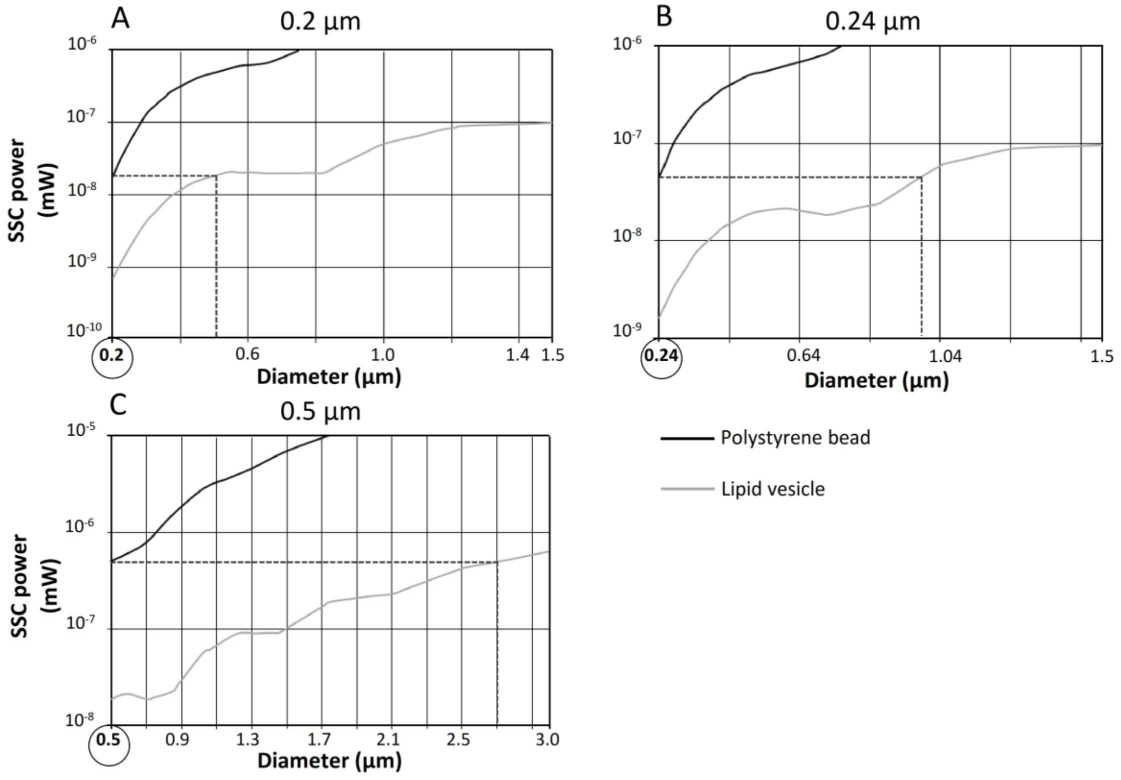

Supplementary Fig. 1. Mie calculations for microvesicle size determination. Mie theory was applied to estimate the side-scattered (SSC) light power of polystyrene beads (black lines) and lipid vesicles (gray lines) in relation to diameter from sizes (A) 0.2 µm, (B) 0.24 µm, and (C) 0.5 µm. The stippled lines mark the lipid vesicle equivalents with estimated SSC light power comparable to that of 0.2 µm, 0.24 µm, and 0.5 µm polystyrene beads. The estimations were performed with the free software Mieplot. The following parameters and values were used: refractive index of lipid vesicles (=1.39), polystyrene beads (=1.59), and medium (~water =1.337). Illumination wavelength (=488 nm) and illumination intensity (=1.09×10^{7} W m^{-2}). SSC collection angle* (31.5-148.5^{o}).
*Taken from FACSAria III (similar optics as the FACSCanto II).

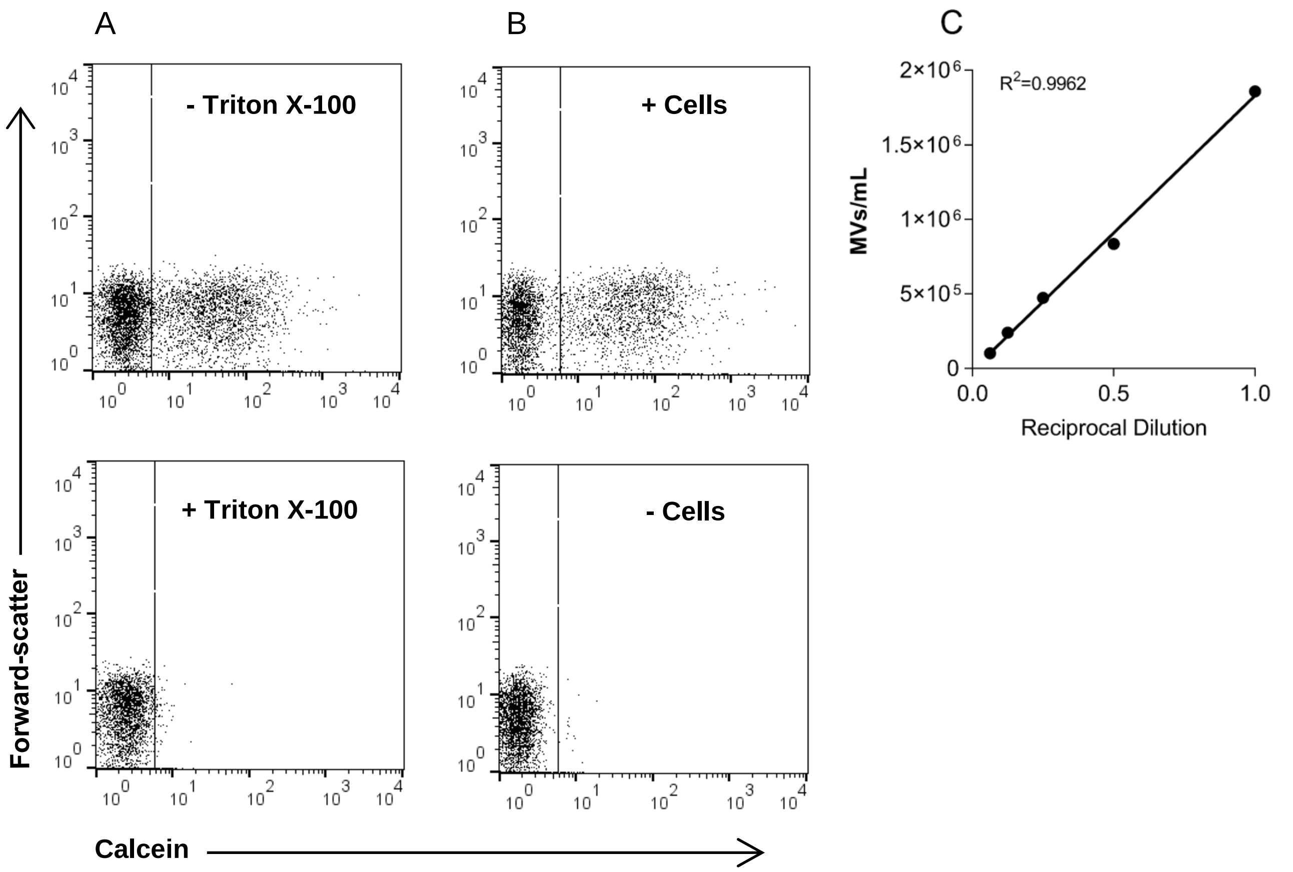

Supplementary Fig. 2. Validation of calcein as a general marker of microvesicles. (A) Treating culture supernatants with detergent (+ Triton X-100) prior to isolation and staining of microvesicles (MVs) abolished the signal from calcein. (B) The calcein signal within the MV gate is negligible in isolates derived from supernatants from wells without added cells (- Cells) relative to corresponding wells with added cells (+ Cells). (C) The potential presence of coincident events was evaluated with serial dilutions of MV-isolates from thawed platelet-poor plasma prior to staining with calcein-AM. MVs in the isolates were quantified and the concentrations (y-axis) were plotted against the reciprocal dilutions (x-axis).
